# Advances in materials used for minimally invasive treatment of vertebral compression fractures

**DOI:** 10.3389/fbioe.2023.1303678

**Published:** 2023-10-25

**Authors:** Pengfei Sui, Tong Yu, Shouye Sun, Bo Chao, Cheng Qin, Jingwei Wang, Erwei Wang, Changjun Zheng

**Affiliations:** Orthopaedic Medical Center, Second Hospital of Jilin University, Changchun, China

**Keywords:** bone cement, minimally invasive surgery, vertebral compression fracture, biomaterial, vertebral implant

## Abstract

Vertebral compression fractures are becoming increasingly common with aging of the population; minimally invasive materials play an essential role in treating these fractures. However, the unacceptable processing-performance relationships of materials and their poor osteoinductive performance have limited their clinical application. In this review, we describe the advances in materials used for minimally invasive treatment of vertebral compression fractures and enumerate the types of bone cement commonly used in current practice. We also discuss the limitations of the materials themselves, and summarize the approaches for improving the characteristics of bone cement. Finally, we review the types and clinical efficacy of new vertebral implants. This review may provide valuable insights into newer strategies and methods for future research; it may also improve understanding on the application of minimally invasive materials for the treatment of vertebral compression fractures.

## 1 Introduction

A vertebral compression fracture (VCF) is defined as a reduction in the height of a single vertebral body by 20% or 4 mm (Black et al., 1999). It is caused by either trauma or a pathological process that causes bone destruction (such as osteoporosis and vertebral tumors). Although osteoporosis is the most common cause of VCFs, tumors, trauma, and infections are also commonly implicated (Liang et al., 2022). Their incidence is usually related to age, occurring in 30% of individuals aged more than 80 years and only 5%–10% of those aged younger (Beall et al., 2018). This condition is becoming increasingly common with aging of the population; approximately 1.5 million individuals are affected each year in the United States. VCF can lead to severe physical limitations including back pain, functional disability, and progressive kyphosis, and ultimately leads to a loss of appetite, malnutrition, and impaired lung function. Recent reports indicate that the thoracolumbar junction (T12 to L2) is the most commonly affected area, accounting for 60%–75% of cases; this is followed by the L2 to L5 region, which accounts for 30% of fractures (Hoyt et al., 2020). Low back pain is therefore the most common clinical manifestation; this severely affects function and quality of life (Ashammakhi et al., 2019). Conservative treatments including medications and physical support cannot offer effective pain control and functional recovery in the long term (Ong et al., 2018). In addition, patients are predisposed to the development of cardiorespiratory complications, which increase patient mortality. Surgery offers an alternative treatment modality for VCF; however, the inability to offer adequate mechanical support often leads to residual postoperative pain. In patients with osteoporotic VCF, surgery is associated with a risk of pedicle screw loosening due to a reduction in bone quality and quantity (Girardo et al., 2019).

Percutaneous vertebroplasty (PVP) and kyphoplasty (PKP) are currently the most common modalities employed for the treatment of VCFs (Hoyt et al., 2020). The procedure involves puncture of the vertebral body with a needle and the injection of cement or other injectable biomaterials via a cannula. This surgical procedure re-stabilizes the height and kyphotic angle of the vertebral body, thereby offering rapid pain relief and an improvement in the quality of life (Roux et al., 2021). Polymethyl methacrylate (PMMA) is widely used in orthopedics and dentistry owing to its high mechanical strength, short setting time, rheological properties, and biocompatibility, making it the most commonly used injectable bone cement for PVP/PKP. However, non-degradability, lack of bioactivity, the presence of unreacted toxic monomers, and the need for high curing temperatures are some of the factors that lead to clinical complications (Martikos et al., 2019). This issue can be partially addressed by using injectable calcium phosphate bone cement (CPC), which has been used as an injectable material in orthopedic surgery due to its chemical similarity to bone and its ability to harden *in situ* (Deng et al., 2020). However, it is brittle and has uncontrollable porosity that does not allow ingrowth of bone; in addition, the paste decomposes when in contact with body fluids and has poor injectability. This led investigators to incorporate certain biomaterials that may enhance its properties (Le Ferrec et al., 2018). In this context, newer materials including magnesium phosphate bone cement (MPC) and calcium silicate (CSC) have been used for minimally invasive treatment due to their unique properties (Huang et al., 2019; Liu et al., 2022). In recent years, vertebral implants composed of implantable materials are being used in third-generation spinal augmentation systems for treating VCFs (Khan and Kushchayev, 2019). The process involves the placement of expandable implants via percutaneous puncture; these are placed either bilaterally or unilaterally through the pedicles. The transition from cement injection alone to the combined use of cement injection and vertebral body implants allows for effective restoration of the height of the collapsed vertebral body; it also improves restoration of vertebral kyphosis and reduces the risk of cement leakage (Moura and Gabriel, 2021). Four vertebral body implant systems are currently in common use; these include the Vertebral Body Stenting, SpineJack, Kiva, and Osseofix systems (Jacobson, 2020; Chang et al., 2021; Gandham et al., 2021; Vendeuvre et al., 2021). Notably, an individualized approach needs to be adopted for implantation; different implants need to be selected for different cases and anatomical locations.

**SCHEME 1:**
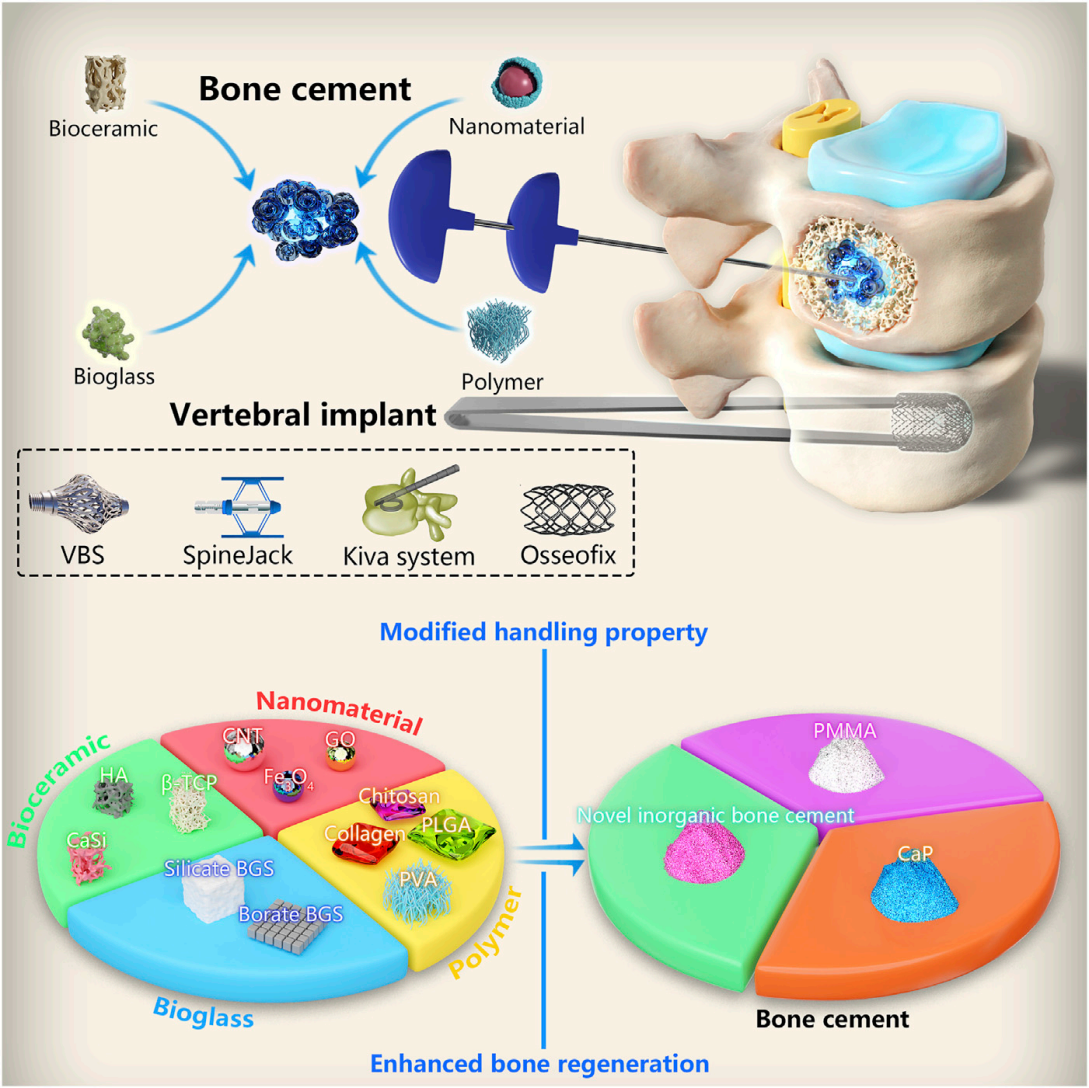
Classification of materials used for minimally invasive treatment of vertebral compression fractures and modification of bone cements.

Materials used for minimally invasive treatment of VCFs have been widely studied in recent years. However, their clinical application is limited by limitations of the materials used. In this review, we describe the various types of bone cement used for minimally invasive treatment of VCFs, starting from the most commonly used material, namely, PMMA; we also discuss their limitations. In addition, we enumerate the materials that are commonly used to improve the properties of bone cement (including bioactive ceramics (Gao et al., 2019), bioactive glass (Golubevas et al., 2017), nanomaterials (Miola et al., 2021), and natural or synthetic polymers (Guo et al., 2021a)) and elaborate on their beneficial impact on the properties of bone cement (Table 1). We additionally describe the types of novel vertebral implants and their clinical efficacy (Scheme 1). Finally, we discuss the prospects for the development of materials used for minimally invasive treatment of VCFs and the directions for further investigation.

**TABLE 1:** Summary of bone cements and modified materials.

Category	Disadvantages	Modifications
Polymethyl methacrylate cement	Excessive mechanical strength	Chitosan, Collagen
	Excessive exothermic reaction	Chitosan, Collagen, Hyaluronic acid
	Low viscosity	Linseed oil, Silk fibroin, Polyvinyl alcohol
	Lack of bioactivity	Chitosan, Collagen, Hyaluronic acid, Bioglass, Bioceramics, Layered double hydroxide
Calcium phosphate cement	Insufficient mechanical properties	Carbon nanotubes, Polyvinyl alcohol, Graphene oxide
	Poor degradability	Chitosan, Hyaluronic acid
	Poor injectability	Collagen, Hyaluronic acid, HPMC
	Poor cohesion	Hyaluronic acid, Starch, HPMC, Chitosan
Calcium sulphate bone cement	Poor washout resistance	Gelatin, Polyvinyl alcohol
	Low compressive strength	Carbon nanotubes, Graphene oxide
	Longer setting time	Silk fibroin, Chitosan
Magnesium phosphate cement	Poor washout resistance	Hyaluronic acid, Gelatin, Chitosan
	Low compressive strength	Bioceramics, Graphene oxide, Bioglass
	Longer setting time	Hyaluronic acid, Chitosan

## 2 Bone cement

Bone cement is widely used in orthopedics and other fields due to the properties of injectability and curing. The treatment of VCF primarily involves the injection of PMMA bone cement into the diseased vertebral body via a minimally invasive surgical approach (Zhu et al., 2020). Notably, other bone cement materials including CPC, MPC, and CSC are being increasingly investigated due to their unique properties.

### 2.1 Classification of bone cement

#### 2.1.1 PMMA-based cement

PMMA, as a representative bone cement, is widely used in the treatment of VCFs due to its outstanding mechanical strength and biocompatibility (Sun et al., 2022b). In 2004, the United States Food and Drug Administration formally approved PMMA bone cement for the treatment of vertebral fractures caused by osteoporosis and tumors. It consists of solid and liquid phases; the PMMA bone cement is formed via an exothermic curing reaction after mixing of both phases. PMMA has become the most widely used material for minimally invasive treatment of VCFs due to its excellent mechanical properties, biocompatibility, and ability to act as a drug-carrying platform (Zheng et al., 2021). Despite its considerable success in clinical applications, it has certain limitations; these include excessive mechanical strength, the occurrence of an exothermic reaction, low viscosity, the lack of osteogenic activity, and the propensity to cause various clinical complications (Sue et al., 2019).

##### 2.1.1.1 Excessive mechanical strength

Owing to the inherent excessive compressive strength and elastic modulus of PMMA, differences in mechanical strength between PMMA and the adjacent vertebral bone may easily lead to fractures in the latter following injection. Finite element analysis suggests that filling the vertebrae with bone cement may significantly alter their stiffness and lead to shifting of load on to the intervertebral disc; this may be responsible for fractures in the adjacent vertebrae (Baroud et al., 2003). Clinical findings have shown that injecting excessive amounts of cement during vertebral kyphoplasty may increase the risks of postoperative vertebral re-fracture (Zhai et al., 2021); in this context, Hu et al. (2019) concluded that injection of more than 40.5% of cement resulted in fractures of the adjacent vertebrae. However, some investigators believe that adjacent vertebral fracture represents a natural evolutionary process in patients with osteoporosis or neoplastic disease, and is not related to the injected PMMA. Notably, several studies have shown that the mechanical properties of PMMA are reduced by mineralized collagen and other materials; clinical results from studies using these cement composites have indicated a significant reduction in the incidence of postoperative adjacent vertebral fractures (from 13.3% to 2%) (Wang et al., 2018a). We therefore believe that it is essential to modify PMMA. In this context, a common approach used for reducing the excessive mechanical strength involves the addition of polymers. The degradation and absorption of these materials lead to the formation of pores within the PMMA and effectively reduce the mechanical strength (Tavakoli et al., 2020).

##### 2.1.1.2 Excessive exothermic reaction

The exothermic reaction associated with polymerization of bone cement can reach temperatures of between 70°C and 120°C, resulting in thermal burns of the surrounding tissue (De Mori et al., 2019). Severe damage to neuromuscular structures have been reported; these can lead to serious complications including paralysis, bleeding, and even death. Bone necrosis and the resultant fiber healing caused by thermal injuries may weaken the interface between the implant and host bone; this may lead to aseptic loosening of the implant (Stoops et al., 2022). Appropriate temperature reduction can be achieved by decreasing the amount of PMMA powder; however, this tends to prolong setting times and reduce the viscosity of bone cement, thereby increasing intraoperative manipulation times and the risk of postoperative cement leakage. The incorporation of biocompatible materials such as polymers, linseed oil, and metamorphic materials may allow absorption of the excess heat generated by polymerization and achieve significant improvement (Lv et al., 2015; Tai et al., 2016; De Mori et al., 2019).

##### 2.1.1.3 Low viscosity

Leakage of bone cement due to low viscosity is a common postoperative complication. Entry into blood vessels can lead to serious consequences including compression of the spinal cord and nerve damage, pulmonary embolism, and cardiac perforation (Hsieh et al., 2019; Naud et al., 2020; Zhang et al., 2022). Guo et al. retrospectively analyzed data from 1,373 patients who underwent PKP and demonstrated significant leakage from the paravertebral venous plexus to be an important risk factor for pulmonary embolism (Guo et al., 2021b). Although symptoms are not observed in most cases, the consequences are often fatal (Hassani et al., 2019). In their retrospective study, Wang et al. found the use of high-viscosity bone cement in PVP/PKP to be a potential option for reducing the risk of leakage (Wang et al., 2022). Similarly, Zhang et al. observed a lower risk of intervertebral disc space or venous leakage with high-viscosity cement (Zhang et al., 2018b). Investigators added polyvinyl alcohol (PVA) to increase the viscosity of PMMA bone cement and prevent cement leakage; the introduction of ethylene via the surface of the PVA membrane allowed the glycidyl methacrylate-PVA membrane to firmly adhere to PMMA-based bone cement. This enabled the cement to covalently react with the PVA membrane (Zhang et al., 2018b). Gelatin particles can also be incorporated to achieve this purpose; notably, particle size and polymer density also regulate cement viscosity (Meng et al., 2013).

##### 2.1.1.4 Lack of bioactivity

As PMMA lacks bioactivity, the major inorganic phases of natural bone are less likely to form on the surface of this polymer; in addition, its surface is not conducive to osteoblast adhesion, proliferation, and differentiation. The formation of a fibrous layer at the PMMA-bone interface prevents direct bone contact, leading to loss of the interface between cement and bone; this further contributes to cement loosening in the postoperative period (Freeman et al., 1982). In their retrospective study, Nakamae et al. found that 25% of patients developed cement loosening after 6 months of PVP; patients with cement loosening had significantly higher mean visual analog scale scores than those without loosening (Nakamae et al., 2018). The incorporation of bioactive materials such as hydroxyapatite (HA) into PMMA represents a good strategy for improving the bioactivity of the latter and enhancing bonding between cement and bone. These materials can allow direct chemical bonding between bone and PMMA cement to enhance interface stability and enhance osteoconductivity (Choi et al., 2010).

#### 2.1.2 Calcium phosphate-based cement

Bone is an organic-inorganic tissue and is composed primarily of collagen and calcium phosphate apatite crystals (Wang et al., 2020a). CPC has a natural affinity for bone tissue, as it resembles the inorganic components of bone. It has become the focus of new developments in injectable bone cement owing to its bioactivity, biocompatibility, osteoconductivity, injectability, and rapid setting time. However, its material properties are relatively immature and there are obvious limitations to its use; investigators are therefore attempting to address these major issues (Klein et al., 2017).

##### 2.1.2.1 Insufficient mechanical properties

Inadequacies in the mechanical property of CPC remain one of the main reasons limiting its application. As per the specifications of International Organization for Standardization 5,833, bone cement needs to have a compressive strength of ≥70 MPa; however, CPC often fails to meet this standard (Schroeter et al., 2020). In addition, the load applied to human bones includes a complex combination of compression, tension, torsion, and bending; parameters such as compressive strength alone often do not accurately reflect the ability of CPC to resist fracture under cyclic loading in clinical settings (Paknahad et al., 2020). As CPC is an inorganic salt material, it is highly brittle; this may make it susceptible to fatigue from cyclic loading and destruction during long-term implantation in the human skeleton. This represents a major limitation that restricts its use in load-bearing sites (Ding et al., 2021). In addition, its low fracture toughness makes CPC considerably sensitive to the presence of defects and imperfections (e.g., porosity); this exacerbates crack propagation. Therefore, the brittleness of CPC and its mechanical properties can be effectively improved by either modifying its microstructure (e.g., porosity and pore size) or activating toughening mechanisms (increasing the resistance to crack extension by using processes such as fiber reinforcement) (Gong et al., 2023).

A denser and more homogeneous matrix composed of smaller crystals is needed to produce smaller pores; this may improve mechanical properties while preserving inward bone growth and other key biological properties. Factors such as porosity, pore size, and particle size of the initial material are key to the mechanical properties of CPC (Lu et al., 2019). In addition to the mentioned effects of densification and homogenization of the cement matrix, the incorporation of fibers or polymers into CPC can enhance mechanical properties. Similar to fiber reinforcement in civil engineering, this is mainly based on three mechanisms: fiber bridging, crack deflection, and friction sliding (Li et al., 2020b). The fibers bridge the cracks to resist their further opening and propagation as the matrix begins to fracture. Crack deflection of the fibers extends the distance over which the cracks propagate; this leads to expenditure of more energy on the newly formed surface. Frictional sliding of the fibers against the matrix during drawing further consumes the applied energy and increases fracture resistance of the composite (Kucko et al., 2019).

##### 2.1.2.2 Poor degradability

CPC is a highly interconnected and porous material; it is almost exclusively composed of micropores and lacks a macroporous structure. The pores in CPCs can be categorized according to their size; micropores have an inner pore width of 100 μm and have a positive impact on the biological response, as they allow protein adsorption, cell attachment, and permeability of the implanted material to body fluids, all of which play a crucial role in promoting osteogenesis. However, the lack of interconnected macropores (exceeding 10 μm) in CPCs hinders angiogenesis and inward tissue growth; it can only be degraded layer-by-layer. This limits degradation at the bone-implant interface and thereby hinders the degradation process (Vezenkova and Locs, 2022). In order to improve CPC degradation, its porosity is controlled by optimizing its material structure. The porosity can be effectively increased by changing the particle size and liquid-powder ratio of the CPC powder phase. The use of powders with smaller particle-size and smaller liquid-to-powder ratios results in ample formation of smaller pores during the crystallization reaction (Lodoso-Torrecilla et al., 2021; Lofrese et al., 2021). Large pores may be introduced into CPC by adding water-soluble and polymeric pore-forming agents (Lu et al., 2021). Polymeric pore-formers are added to the CPC paste as a second solid phase. After curing of the CPC, these polymeric pore-formers begin to degrade and produce macroporous CPC composites; notably, these polymers confer unique properties, such as improved osteogenesis. In this context, CPC should ideally degrade at an appropriate rate to allow for concomitant new bone formation.

##### 2.1.2.3 Poor injectability

The poor injectability of CPC is one of the main factors that limit its use in PVP and PKP procedures. The higher liquid content in uncured CPC leads to lower viscosity, lower cohesion, increased setting times, and lower mechanical strength. This subsequently leads to extravasation of bone cement from the surgical site, leading to complications such as pulmonary embolism. This possible deviation of the actual composition of the extruded paste may be attributed to the filtration pressure, which affects the injectability of CPC pastes (Habib et al., 2008). During the construction of an injectable model, Bohner and Baroud found that the reduction of extrusion pressure (by increasing fluidity) and permeability of the paste improved cement injectability. Certain changes were made to the cement to address these two issues; the average particle size was reduced, the liquid-solid ratio was increased, round and de-agglomerated particles were used, a wide particle size distribution was adopted, ions or polymers were added to minimize particle interactions, and the viscosity of the mixture was increased (Bohner and Baroud, 2005). Ishikawa et al. observed that CPC pastes made from round particles could be injected more easily than those made from irregular particles (Ishikawa and Asaoka, 1995). However, in view of the favorable effect of high viscosity on resistance to disintegration, the latter two strategies appear to be the most appropriate for improving cement injectability. The addition of binders may also effectively reduce phase separation; however, this can result in several undesirable consequences, such as an increase in the force required for injection and a decrease in mechanical properties (Schickert et al., 2020b).

##### 2.1.2.4 Poor cohesion

Cohesion represents the ability of CPC to harden and maintain the integrity of the cement paste in a static aqueous environment without disintegrating into small particles; it prevents attrition of the paste by the surrounding liquid (Vezenkova and Locs, 2022). The cohesion of CPC paste often depends on the forces between the constituent particles and the interaction between the paste and the surrounding fluid. Spatial stabilization is usually associated with the presence of dissolved polymers on the surface or space between particles. Thus, the addition of polymers can spatially stabilize the cement paste and increase cohesion. Increasing the viscosity of the mix is another effective approach for increasing cohesion. Numerous biopolymers, including hydroxypropyl methylcellulose and starch, have been blended into powders or liquids of CPC (Liu et al., 2014; Tian et al., 2021). Small amounts of these biopolymers can significantly improve cohesion and erosion resistance of CPC. However, although these viscous solutions may significantly improve paste cohesion, they may affect the setting time and mechanical properties in certain cases.

#### 2.1.3 Novel inorganic bone cement

The study of magnesium phosphate for bone cement is a relatively recent development. The excellent osteogenic and vasculogenic properties of MPC are the key factors that contribute to its use in research; these factors are mainly attributed to magnesium ions, which exert an osteogenic effect via the activation of osteoblast activity. They promote the proliferation of osteoblasts via the mitogen-activated protein kinase/extracellular signal-regulated kinase signaling pathway by increasing phosphorylation of the latter (thereby enhancing the level of c-FOS) and inducing the phosphorylation of glycogen synthase kinase-β (thereby enhancing the level of β-conjugated proteins) (Wang et al., 2018b). Magnesium ions also promote osteogenesis by promoting angiogenesis (Zhang et al., 2021). Recent studies have found that depletion of magnesium content is associated with low bone mineral density, reduced bone progression, the development of osteoporosis, and skeletal improvement; they have also found that higher magnesium intake effectively inhibits a reduction in bone mineral solids in patients with osteoporosis. In this context, a magnesium alloy was found to release magnesium ions after implantation in osteoporotic rats; this increased bone morphogenetic protein 2-related osteogenesis and reduced the deleterious effects of osteoporosis (Guo et al., 2013). The localized release of magnesium ions from magnesium implants in an animal model of osteoporosis was also found to contribute to the formation of a condensate around the implant; significantly higher volumes of new bone were observed in magnesium-containing specimens (Galli et al., 2018). Although MPC does not currently meet the criteria for clinical application in terms of handling properties, it can be improved considerably by employing a wide variety of modifications. Its mechanical properties allow it to withstand loads of up to 112 MPa; this is equivalent to the mechanical strength of human cortical bone (Liu et al., 2022). Liu et al. demonstrated the acceptable injectibility and filling properties of MPC; they injected MPC into 3 dimensional-printed artificial vertebral bodies and porcine spine models and were able to achieve a good distribution. This could allow it to be successfully used in PVP/PKP or for filling other bone defects (Liu et al., 2023). Chitosan has been added to MPC to improve its handling properties; chitosan-MPC has a longer setting time, lower reaction temperature, higher strength, and more neutral pH than MPC (Yu et al., 2020).

CSC has also been used in orthopedic applications due to its superior biocompatibility and bone regeneration properties, which are similar to those of MPC. CSC generates less heat during the exothermic curing reaction and demonstrates superior bioactivity, excellent osteoconductive activity, and degradability. Huang et al. proposed a method to synthesize biodegradable calcium silicate cement by incorporating strontium into cement through solid-state sintering. The degradation rate of the cements increased with increasing content of strontium, consequentially raised the levels of released strontium and silicon ions. The elevated dissolving products may contribute to the enhancement of the cytocompatibility, alkaline phosphatase activity and osteocalcin secretion (Huang et al., 2019). Furthermore, the MPC/CS composite bone cement demonstrated apatite mineralization ability and osteogenic potential. This composite was experimentally shown to stimulate the proliferation of MC3T3-E1 cells (Liu et al., 2022). However, it demonstrates poor scour resistance, deficiencies in mechanical properties, and long curing times. These deficiencies limit its application as a clinical material and warrant modification (Zanfir et al., 2019).

## 3 Materials used to improve bone cement

### 3.1 Bioceramics

**FIGURE 1:**
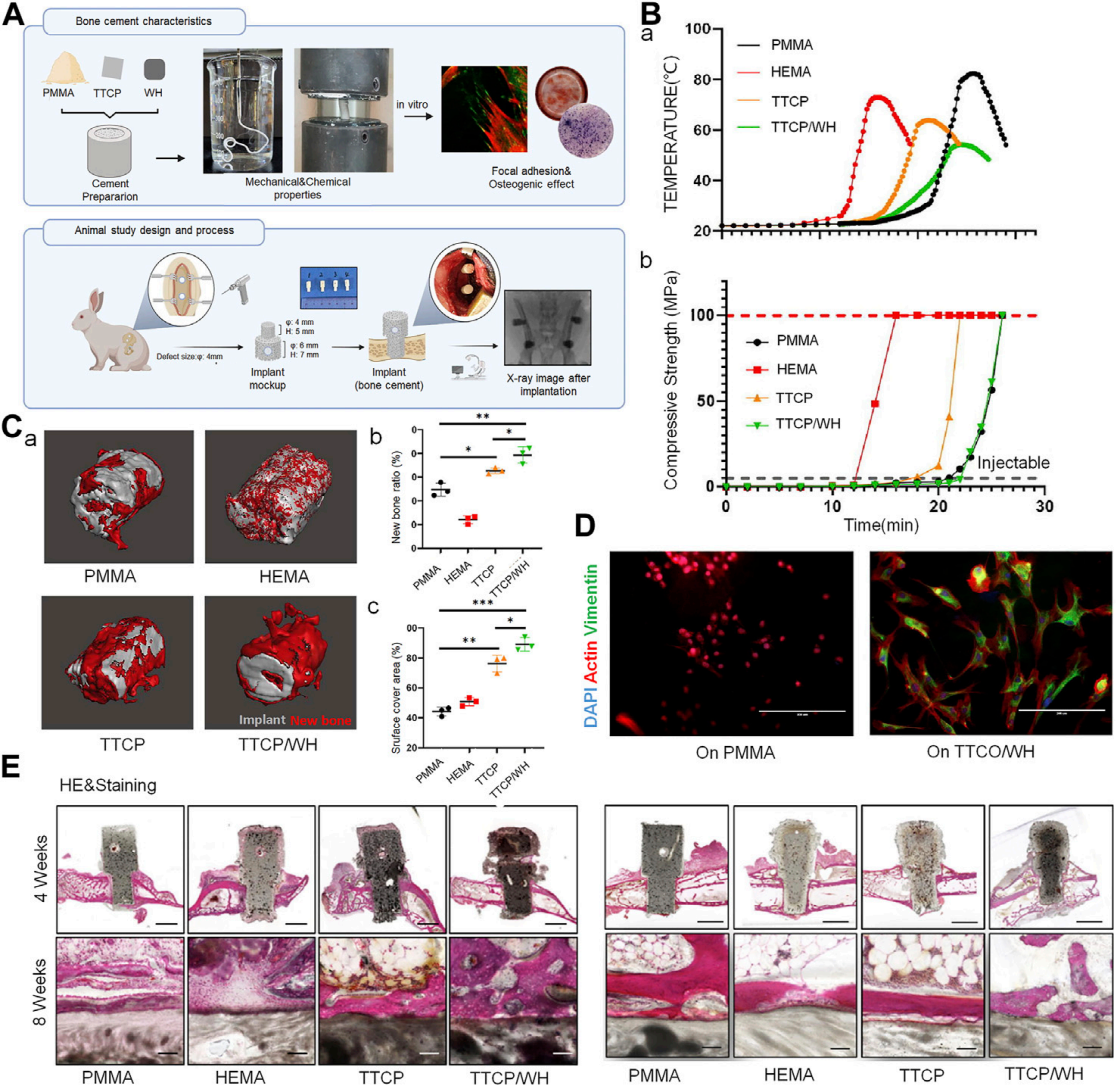
Bioactive ceramics as bone cement additives for enhancing osseointegration and bone regeneration. **(A)** Bone cement preparation and study design of *in vitro* and *in vivo* models. **(B) (A)** Temperature measurement of cement from mixing to setting. **(B)** Compressive strength measurement of cement during reaction. **(C)** (a) New bone formation around implanted scaffolds. (b) New bone generation ratio and (c) new bone covered area of cement scaffold at 4 weeks after implantation into bone defect. **(D)** Actin, vimentin and DAPI immunostaining of BMSCs after seeding on cement scaffold. **(E)** Representative histological findings from implant sites at 4 weeks (sagittal plane) and 8 weeks (coronal plane) post-implantation (the scale bar = 4 mm and 100 μm); hematoxylin and eosin stain. ^©^ 2023 The Authors. Published by Elsevier Ltd.

Bioactive materials are defined as those that stimulate a beneficial response in the body, especially in terms of binding to host tissues (Jones, 2013). Bioactive ceramics and glass have been added to bone cement due to their superior biocompatibility and osteogenic capacity. Traditional bioactive ceramics such as HA, β-tricalcium phosphate (TCP), and calcium silicate ceramics have been widely used in the modification of bone cement (Liu et al., 2023b) (Figure 1).

HA has been used as an inorganic filler in bone cement to improve its biocompatibility, as it has a chemical composition and crystal structure similar to that of apatite found in human bone tissue. HA is highly osteoinductive; this promotes chemical interactions with osteoblasts and the local microenvironment and thereby promotes the formation of new bone (Thorfve et al., 2014). In terms of processing properties, researchers have extensively investigated the feasibility of combining HA/brushite with PMMA. Aghyarian et al. prepared two composite bone cements, namely, HA-PMMA and brushite-PMMA, and found that the addition of both materials increased cement viscosity. These cements also exhibited high shear thinning, which aided injection (Aghyarian et al., 2014) and an appropriate increase in compressive strength. They subsequently prepared bone cement with various concentrations of brushite; PMMA was replaced by a 40% mass concentration of brushite to prepare dual solution cement, which could provide an optimal combination of the studied properties. The cement was viscous, highly injectable, and had high compressive strength (Rodriguez et al., 2014). Further characterization was performed in porcine vertebral bone and in two functional cadaveric spinal units, where the biomechanical properties of calcium phosphate-PMMA were found to be comparable to those of commercial bone cement; this indicated excellent prospects for clinical application (Aghyarian et al., 2015; Aghyarian et al., 2017). Although β-TCP demonstrates a good dissolution rate and bone regeneration capacity, the mechanical strength of TCP/PMMA composite bone cement was found to be lower than that of its conventional counterpart; it therefore failed to meet the enhancement requirements of clinical implants (Yang et al., 2015). Biphasic calcium phosphate, a mixture of β-TCP and HA, can effectively harmonize the properties of both materials. Its implantation improves biodegradability of TCP, leading to supersaturation of the local microenvironment with calcium and hydrogen phosphate; this accelerates the formation of calcium-deficient HA microcrystals, ultimately promoting mineralization of the extracellular matrix and subsequent generation of new bone during healing (Zhang et al., 2018a).

Calcium silicate bioceramics are being increasingly investigated as potential novel bioceramic materials for bone grafting, as their osteogenic properties are superior to those of HA (Vallet-Regi and Arcos, 2005). Several studies have shown that in a physiological environment, silicon ions released from calcium silicate ceramics play an important role in promoting bone regeneration by stimulating the proliferation of mesenchymal stem cells, osteogenic differentiation, and osteoblastic gene expression (Lin et al., 2015). In their study using a goat vertebral defect model, Sun et al. used new PMMA/calcium silicate hybrid cements for PVP and PKP; these cements optimally filled and stabilized vertebral defects and significantly promoted new bone formation in defective vertebrae at 6 months after injection (Sue et al., 2019). However, silicate bioceramics offer insufficient mechanical strength due to degradation. A series of silicate-based bioceramics have therefore been developed, including those that incorporate iron, magnesite, akermanite (Ca_2_MgSi_2_O_7_, AKT), and tremolite (CaO-MgO-2SiO_2_) (Chen et al., 2015b; Choudhary et al., 2020). The compressive strength of akermanite/PMMA composite bone cement is approximately 100 MPa, which is comparable to that of commercial PMMA bone cement (at 73–120 MPa) (Chen et al., 2015b). Notably, the compressive strength and Young’s modulus of PMMA-diopside composites match the lower limit of those of cancellous bone (Choudhary et al., 2020).

### 3.2 Bioglass

**FIGURE 2:**
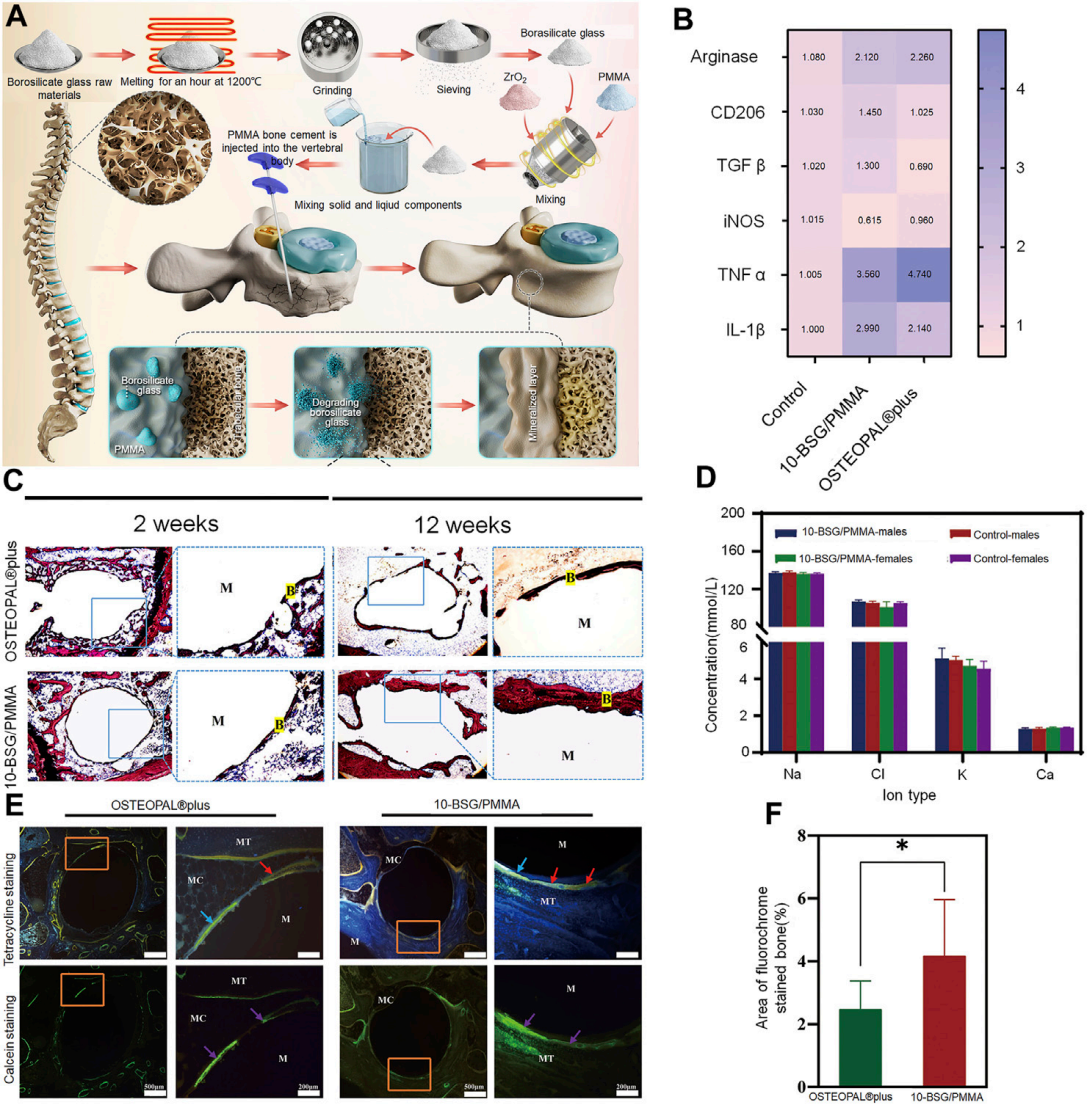
Borosilicate glass (BSG)-reinforced PMMA bone cement used for vertebroplasty. **(A)** Schematic diagram illustrating the preparation of BSG/PMMA cement and the its use for promoting bone repair. **(B)** Expression of pro-inflammatory genes and anti-inflammatory genes after culture with 10-BSG/PMMA or OSTEOPAL^®^ plus cement for 3 days. **(C)** Van Gieson staining of rat tibia defects after 2 and 12 weeks of cement implantation. The images on the left and right are from the same group; they represent the overall picture and the partial images. **(D)** Results of subchronic systemic toxicity in the experimental and control groups, including rat serum electrolyte indices at different time points. **(E)** Overall and partial images of sequential fluorescence staining, and **(F)** semi-quantitative evaluation of new bone formation based on calculation of the area with fluorescent staining (as determined from panel **E**). Results are shown as means ± standard deviation (*: *p* < 0.05). Copyright ^©^ 2022, American Chemical Society.

Bioactive glass (BG) includes glass that can produce a specific biological response at the material-bone interface and promote efficient bonding between them (Shearer et al., 2023). Silicate and borate BGs are the most commonly used. Notably, BGs bond to bone faster than other bioceramics (Zhang et al., 2022a) (Figure 2). The process of osseointegration begins with the release of silica ions from the surface of the BG; the released ions form a layer of silica on the surface and then form an amorphous calcium phosphate precipitate, which initiate the formation of a HA layer that bonds to the bone cortex, further activating cell migration and triggering new bone formation (Cole et al., 2020a). Its osteogenic properties are an area of considerable interest as its dissolution products stimulate bone progenitor cells at the genetic level (Rahaman et al., 2011). BG stimulates bone formation by polarizing macrophages from the M1 to M2 phenotype and thereby increasing the activity of relevant genes. M1 macrophages produce pro-inflammatory cytokines and exhibit strong microbicidal properties in the early stages of inflammation. However, in the later stages of bone regeneration, persistent and excessive inflammation hinders complete local bone tissue remodeling. M2 macrophages produce anti-inflammatory cytokines to promote the tissue healing process. Therefore, regulation of the transition from M1 to M2 phenotypes represents a crucial step in the process of bone regeneration (Gomez-Cerezo et al., 2018). In this context, an alkaline environment may enhance bone formation in osteoporosis by inhibiting osteoclast activity and increasing osteoblast viability. A moderately alkaline pH of 7.8–8.5 has been reported to provide a favorable environment for new bone formation (Ding et al., 2023). Numerous researchers have prepared novel BGs that release boron and strontium ions, which create an alkaline environment; this is of particular significance in the treatment of osteoporotic VCFs. In their study on osteoporotic rabbits, Chen et al. constructed a porous injectable composite by combining silicate BG with PMMA; they found that injection of the composite into the vertebrae of the rabbits increased the bone volume fraction (trabecular bone to total bone volume) from 28.27% ± 1.69% to 38.43% ± 1.34% (Chen et al., 2015a). Hu et al. also reported similar findings in an osteoporotic rabbit model. The application of BG-modified CPC to the bone defects resulted in significant upregulation of osteogenic marker expression (including Runx2, alkaline phosphatase, osteopontin, and osteocalcin) and a significant decrease in osteoblastic marker expression (including tartrate-resistant acid phosphatase, matrix metalloprotease 9, and histone K) in the osteoblasts (Hu and Xu, 2019).

Although numerous experiments have demonstrated that the addition of BG sufficiently enhances the bioactivity of bone cement, it impacts the mechanical properties (including injectability) of the cement. In this context, borate-based BG demonstrates a controlled degradation rate; the bioactivity and degradation rate of borosilicate-based BG can be regulated by the introduction of variable quantities of boric oxide to match the growth rate of new bone tissue (Cui et al., 2016). In their study, Cole et al. added borate-based BG to PMMA; long-term dissolution of BG could be achieved without affecting short-term degradation. Ion release was also maintained without affecting mechanical strength. The compressive properties remained higher than those required by the American Society for Testing and Materials and International Organization for Standardization (Cole et al., 2020b). BG also enhances the compressive strength of CPC, as it becomes smaller and denser after pore sintering; this increases the compressive strength of HA/BG composites. This increase improves its load-bearing capacity and implant stability in the tissue (Ebrahimi and Sipaut, 2021). BG also improves the initial and long-term compressive strength of calcium sulfate bone cement and demonstrates good injectability and controlled setting times; all of these make it suitable for vertebral augmentation (Mansoori-Kermani et al., 2023). However, the introduction of BG adversely affects the handling properties of bone cement to varying degrees. A decrease in BG particle size has been found to reduce injectability of the cement. The fine BG particles agglomerate and absorb more water; this increases the friction between them. The setting time of formulated cement also increases significantly with a decrease in BG particle size, as the cohesion in the cement paste weakens (Hasan et al., 2019; Mabroum et al., 2022).

### 3.3 Nanomaterials

#### 3.3.1 Carbon nanotubes

Carbon nanotubes (CNTs) can significantly improve the mechanical properties of bone cement. This may be mainly attributed to certain unique properties including nanoscale diameters, longer length, higher strength and stiffness, and considerably high aspect ratios. Multi-walled CNTs prevent cracks in the cement from expanding; they provide a bridging effect at the tails of the crack in a direction perpendicular to that of crack expansion. In their study, Sadati et al. found that the incorporation of 0.5% of multi-walled CNTs into PMMA significantly increased tensile strength, elastic modulus, and bending strengths by 37%. The finite element method was used to simulate the bridging mechanism of PMMA/multi-walled CNT nanocomposites (Sadati et al., 2022). Combining the functionalized CNTs with PMMA significantly reduces the high polymerization temperature of PMMA. The reduction in generated heat translates to a reduction in the thermal necrosis index value of the corresponding nanocomposite cement; this may reduce the high temperatures *in vivo* and decrease the possibility of heat-induced bone tissue necrosis induced by polymerization of PMMA cement. In their study, Ormsby et al. found that the addition of functionalized multi-walled CNTs led to a significant reduction in the quantity of heat generated by the exothermic polymerization reaction of PMMA bone cement; it also significantly reduced thermal necrosis index values from 3% to 99% (Ormsby et al., 2011). Mabroum et al. had combined CNT with commercial bone cement; they also observed a significant reduction in the heat generated by the exothermic polymerization reaction of Simplex PTM bone cement. They suggested that the carboxylated multi-walled CNTs acted as a heat trap in the bone cement matrix to reduce the generated heat (Ormsby et al., 2014).

**FIGURE 3:**
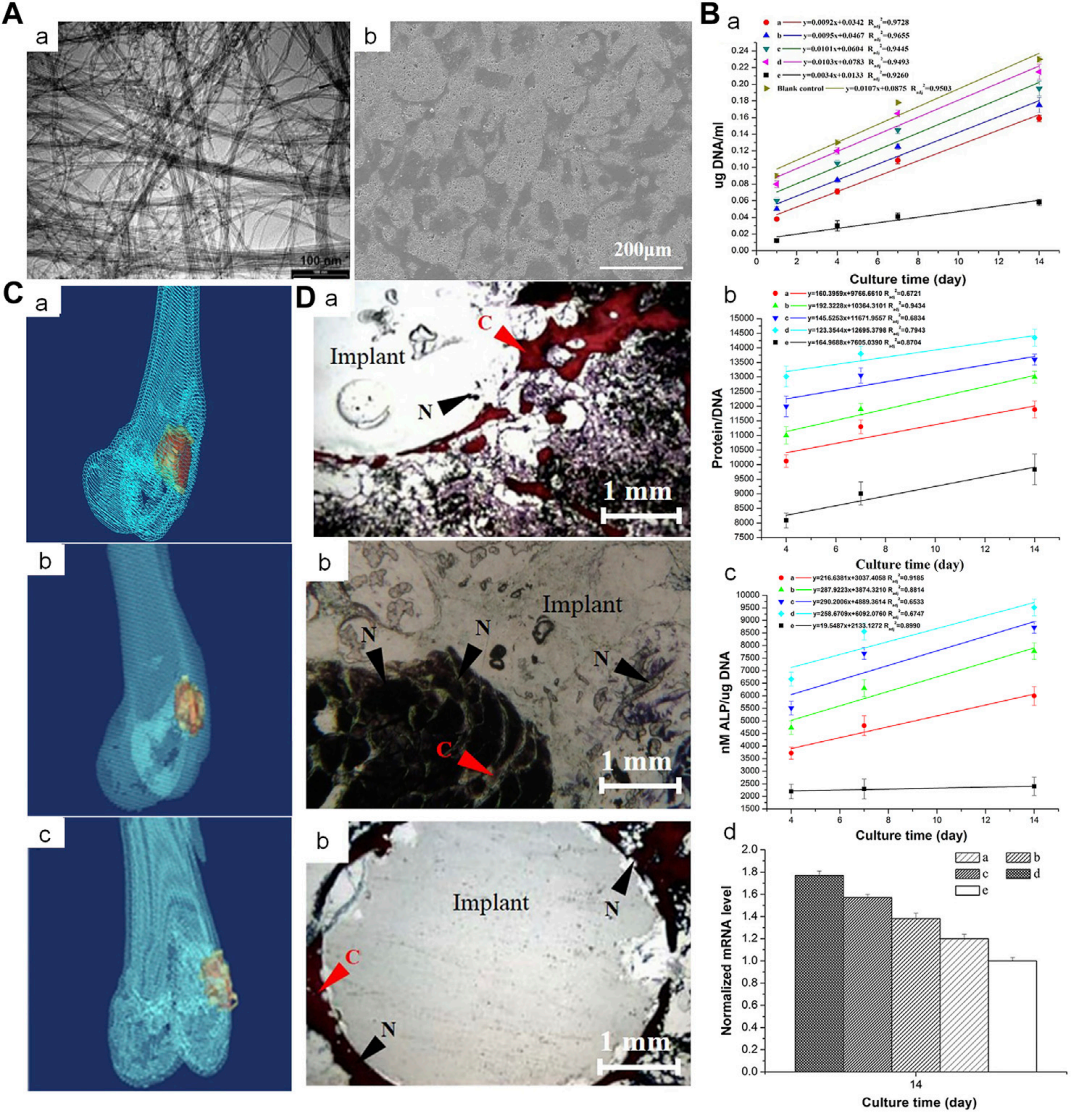
Incorporation of multi-walled CNTs into PMMA bone cement improves cytocompatibility and osseointegration. **(A)** (a) Transmission electron microscopy image of NC3151 grade multi-walled CNT. (b) Scanning electron microscope images of PMMA bone cement loaded with multi-walled CNT powder affecting the proliferation of rabbit BMSCs. **(B)** (a) deoxyribonucleic acid (DNA) content, (b) Protein/DNA content and (c) ALP/DNA (mean ± SD) of the rabbit BMSCs when exposed to blank control and PMMA bone cements loaded with different concentrations of multi-walled CNT powder. **(C)** 3-dimensional reconstruction of computed tomography images of PMMA-multi-walled CNT bone cement specimens from Group D after 4, 8, and 12 weeks. **(D)** Van Gieson-stained images of PMMA-MWCNT bone cement specimens from group D (1.0 percent by weight) after 4, 8, and 12 weeks: (a) 4 weeks, (b) 8 weeks, (c) 12 weeks. Collagen fibers **(C)** are stained red. The nucleus (N) is stained brown-black. Crown Copyright ^©^ 2019 Published by Elsevier B.V. All rights reserved.

The possibility of nanotoxicity needs to be considered in the case of CNT-based biomaterials (Wang et al., 2019) (Figure 3). Pahlevanzadeh et al. incorporated CNT into PMMA-monticellite bone cement; they found that the cement retained good bioactivity after incorporation of CNT, as evidenced by the absence of cytotoxic effects in MG63 cells (Pahlevanzadeh et al., 2019). Similarly, the addition of CNT to MPC exerted no cytotoxic effects; the cells exhibited appropriate adhesion to the bone cement and acceptable proliferation (Esnaashary et al., 2020). However, Medvecky et al. reported conflicting results; their experiments, that involved material testing and live/dead staining of CNT-CPC, suggested that multi-walled CNT composite cement surfaces were cytotoxic (Medvecky et al., 2019). Notably, some believe that an optimal CNT content is required for cellular activity. An increase in the concentration of CNT in PMMA nanocomposites allows for adequate survival and proliferation of mesenchymal stem cells (MSCs) on their surface; the cell density decreases significantly when the number of CNTs exceeds 0.25 percent by weight (Sadati et al., 2022).

#### 3.3.2 Graphene oxide

As graphene has a lower metal impurity content than CNTs, the purification processes for removal of trapped nanoparticles require less time. Based on the method of fabrication, graphene can be categorized into numerous subtypes; these include graphene, graphene oxide (GO), and reduced GO, which is of particular interest. GO has a larger number of hydrophilic groups than simple CNT and graphene; these allow it to form chemical bonds (between functional groups) with molecules in the bone cement. This facilitates good dispersion, thereby enhancing mechanical strength and improving biological properties. In their study, Paz et al. loaded different proportions of graphene and GO onto PMMA; they found that the PMMA cement with a lower load of graphene or GO powder (≤0.25 percent by weight) showed significantly superior fracture toughness and fatigue properties (Paz et al., 2017). In addition, GO powder demonstrated greater dispersion and improvement in mechanical properties than those obtained with graphene powder. In their study, Arici et al. compared the mechanical properties and cellular activity of CNT with those of GO. They found that GO further improved the percentage of cell viability, conferred superior mechanical properties, and offered a more stable pH and cell viability than CNT; this may be attributed to the larger surface area of GO (Arici et al., 2023). The introduction of GO has also been found to improve cell viability and osteogenic differentiation. All anabolic genes including *COL1A1*, *BMP4*, *BMP2*, *RUNX2*, and *ALP* demonstrate stimulatory effects, while catabolic genes (*MMP2* and *MMP9*) exert inhibitory effects (Mirza et al., 2019). Another mixing approach involves the incorporation of hybridized GO/CNT into bone cement. This approach offers better dispersion properties than CNTs and GO alone; it also shortens the final setting time and reduces the mobility of MPC. In this context, a study showed that the addition of 13.77% GO/CNTs (by weight of cement) increased the compressive and flexural strength of MPC by 17.50% and 0.05%, respectively (Du et al., 2020).

#### 3.3.3 Magnetite (Fe_3_O_4_)

**FIGURE 4:**
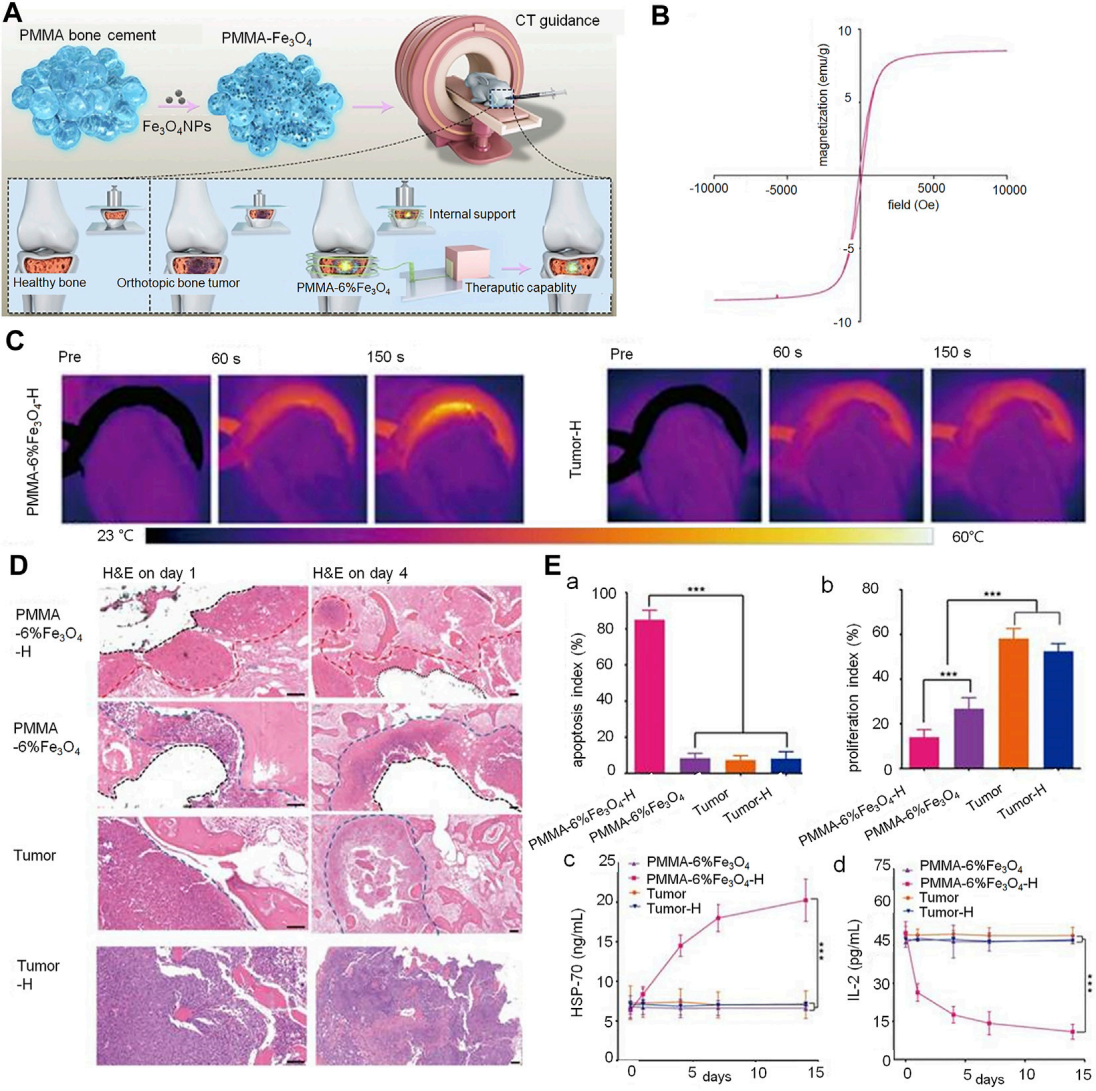
PMMA-Fe_3_O_4_ composite used for internal mechanical support and magnetic thermal ablation of bone tumors. **(A)** Prepared magnetic PMMA bone cement for magnetic thermal ablation of tumors. **(B)** Magnetic hystersis loop of polymerized PMMA-6% Fe_3_O_4_. **(C)** Thermal images of rabbit leg in the PMMA-6% Fe_3_O_4_-H group and Tumor-H group. **(D)** Hematoxylin and eosin staining on day 1 (scale bar: 50 μm) and day 4 (scale bar: 100 μm) (red dotted line: edge of ablation, blue dotted line: edge of the tumor, black dotted line: edge of removed PMMA-6% Fe_3_O_4_ composite). **(E)** (a) and (b) indicate the apoptosis index (AI) and proliferation index (PI) of each group. (c) Heat shock protein-70 levels of rabbit serum in different groups before and after magnetic thermal ablation. (d) Interleukin-2 levels of rabbit serum in different groups before and after magnetic thermal ablation. Copyright ^©^ 2019, Ivyspring International Publisher.

The combination of nanomaterials with PMMA represents an important area of interest in the treatment of tumor-induced VCFs. While the PMMA implanted in the fracture site plays a role in supporting the fractured vertebrae and relieving pain, the magnetic nanomaterials act as heat-seeded materials in magnetic thermotherapy; they generate heat due to loss of magnetism in the presence of an external magnetic field (Yu et al., 2019) (Figure 4). Heating of the cancerous area to temperatures of over 42°C kills the cancer cells, while allowing normal cells to survive. Magnetite is well suited for use as a heat seed material due to its excellent heat-generating properties and biocompatibility. In their study, Ling et al. placed PMMA-Fe_3_O_4_ in an *ex vivo* magnetic field; the increase in temperature of resected bovine liver was found to positively correlate with the iron content and time. This suggested that the intratumoral temperature is controllable (Ling et al., 2017). However, as the heat generated by magnetothermic materials may damage surrounding healthy tissues (especially the spinal cord), Harabech et al. evaluated the heating effect of Fe_3_O_4_ nanomaterials in bovine vertebrae in an *ex vivo* alternating magnetic field. The temperature in the PMMA-magnetic nanoparticle composite rose by approximately 7°C; however, that in the spinal column only rose by only 1°C, thereby creating a smaller thermal impact on the spinal cord (Harabech et al., 2017). Although Fe_3_O_4_ demonstrates excellent heat generation properties as a magneto-thermal material, its rate of warming and bioactivity need to be improved. Certain investigators have attempted to increase the weight percentage of magnetic nanoparticles in PMMA in order to improve the magneto-thermal efficiency of nanomaterials. However, an inappropriately high weight ratio of magnetic nanoparticles affects the physicochemical properties and increases cytotoxicity (Miola et al., 2021). Ren et al. attempted to improve performance by adding 1 percent by weight of Zn_0_.3Fe_2_.7O_4_ nanoparticles to PMMA; in addition to providing reliable mechanical support, the resulting bone cement demonstrated high thermal efficiency (Ren et al., 2022). The wrapping of Fe_3_O_4_ with GO (which has superior thermal conductivity) addresses the important issue of non-uniform heating of magnetic thermal materials; this allows for more rapid heating of the composite materials and achievement of thermal equilibrium. This shortens the time of thermal therapy and reduces heat-resistance caused by an excessively long heating time (Yan et al., 2019).

#### 3.3.4 Other nanomaterials

Layered double hydroxide has recently provoked considerable interest owing to its excellent properties. Liquid MMA monomer was added to the pre-polymerized PMMA and powders of COL-I and/or LDH, and the polymerization reaction of MMA was initiated at room temperature (25°C) for 20 min after the powders were thoroughly mixed. It demonstrates outstanding thermal insulating properties, which may inhibit thermal diffusion during the polymerization reaction of methylmethacrylate and help protect the surrounding osteoblast-associated cells. In addition, the magnesium ions released by LDH promote osteogenesis. The larger micro sheets of layered double hydroxide are able to produce a certain number of holes on the surface of PMMA; this is beneficial for osseointegration between cement and bone (Wang et al., 2021). Titanium dioxide and magnesium oxide nanoparticles have also been added to PMMA due to their excellent osteogenic activity (Li et al., 2020a; Vedhanayagam et al., 2020).

### 3.4 Polymer materials

#### 3.4.1 Natural polymers

Natural polymers are used as biomaterials in medicine due to their excellent biomimetic properties and biocompatibility (Guo et al., 2021c). Chitosan, a linear polysaccharide obtained by deacetylation of chitin, is one of the most common natural polymers used (Sultankulov et al., 2019). Given its excellent biocompatibility, biodegradability, and bioactivity, it has been used for the modification of bone cement. As chitosan is degraded *in vivo*, its incorporation into PMMA bone cement creates appreciable porosity. The increase in porosity facilitates osseointegration between bone and cement and promotes more stable fixation; it also reduces the mechanical strength of PMMA-based bone cement, thereby reducing the difference with bone (Sun et al., 2022a). The improvement in bioactivity and polymerization temperature were found to be dose-dependent; an increase in the loading concentration of chitosan (to >10%) significantly reduced the heat generated by PMMA during polymerization (De Mori et al., 2019). In their study, Zapata et al. obtained similar outcomes compared to CS when using <15% loading; the composite PMMA/CS bone cement having >15% loading achieved more rapid deposition of calcium and phosphorus ions, and showed more rapid bioactivity (Valencia Zapata et al., 2020). Incorporating different forms of chitosan may also produce different effects. In their study, Zamora Lagos et al. incorporated different forms of chitosan into PMMA; CS sheets provided greater porosity to the cement than CS spheres (Zamora Lagos et al., 2020). However, CS microspheres demonstrated greater degradation in bone cement, thereby effectively improving the osteoconductivity and degradation of CPC (Meng et al., 2019).

**FIGURE 5:**
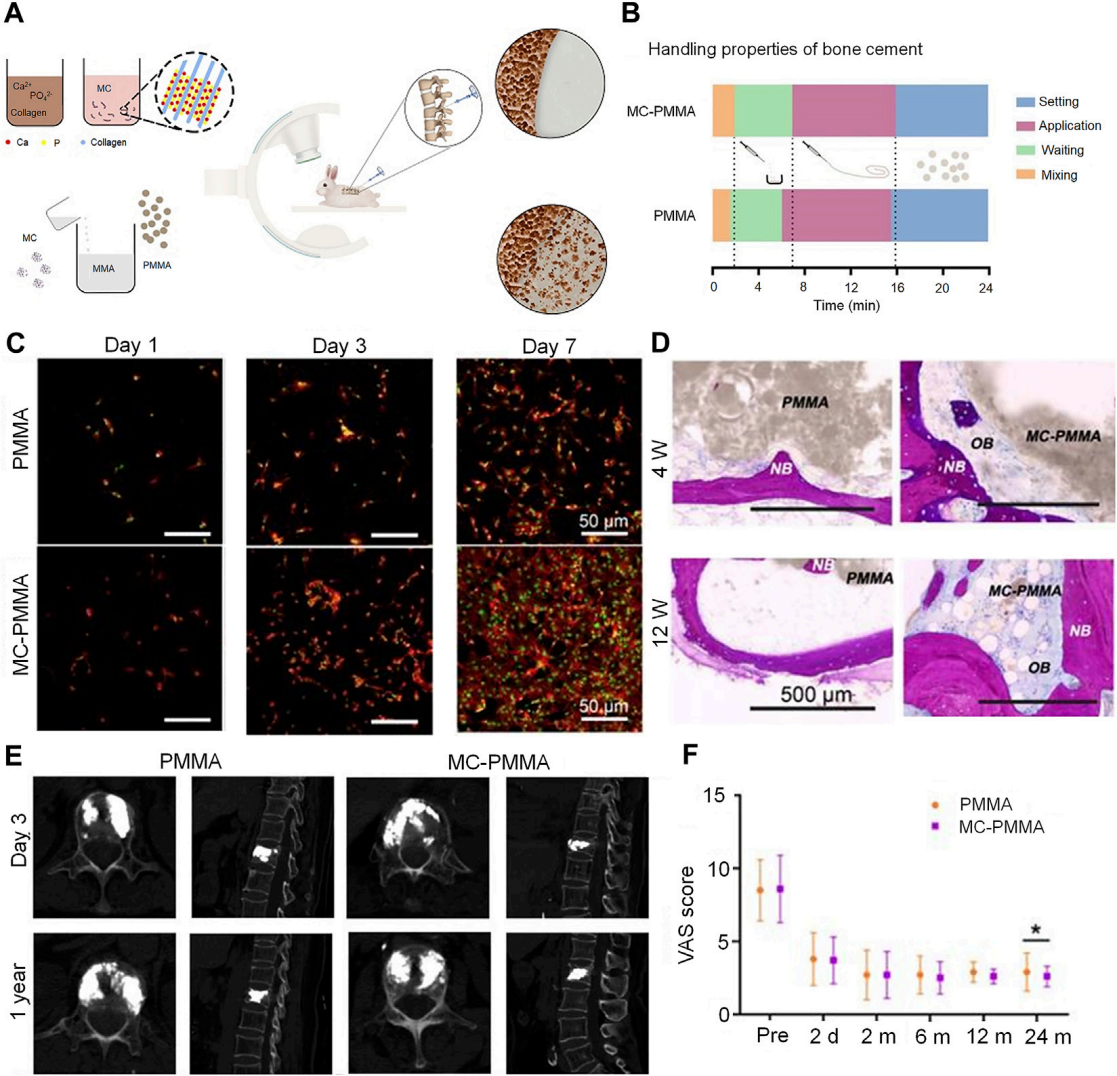
Mineralized collagen-reinforced PMMA bone cement for the treatment of osteoporotic vertebral compression fractures. **(A)** Preparation of mineralized collagen and PMMA bone cement. **(B)** Handling properties of bone cements. **(C)** Morphology of BMSCs on days 1, 3, and 7 with MC-PMMA or PMMA bone cement. Cells stained with rhodamin-phalloidin for F-actin (red) and SYTOX Green for nuclei (green). **(D)** Histological staining in the PMMA and MC-PMMA groups after 4 and 12 weeks using methylene blue (light blue) and basic fuchsin (red). The bone cement is in gray. **(E)** Lateral projection re-examination by computed tomography at 3 days and 1 year after surgery. **(F)** The visual analog scale score was evaluated by three doctors. Results are presented as the mean ± standard deviation; *p < 0.05. Copyright ^©^ 2020, Ivyspring International Publisher.

Collagen is a natural antigenic biomaterial found in the skin, ligaments, bone, and cartilage (Li et al., 2021). Type I collagen accounts for 90% of the total collagen and is present in large quantities in the bone extracellular matrix secreted by osteoblasts. Collagen can be easily combined with other biomaterials; mineralized collagen (MC) can be formed by mineralization of HA and collagen molecules. Owing to similarities in structure and chemical composition between MC and natural bone components, the former demonstrates good osteogenic activity; it also increases the differentiation of MSCs to osteoblasts (Zhu et al., 2023). MC-modified bone cement significantly improves the adhesion of preosteoblasts and their proliferation; this promotes good osseointegration between the cement and host bone tissue. It also promotes higher alkaline phosphatase activity (secreted by human bone marrow MSCs) and higher expression of osteoblast-specific genes (Jiang et al., 2015). Notably, osteogenic differentiation has been found to be more than twice as high in MC-PMMA than in PMMA after 21 days of culture (Wu et al., 2016). In addition, the introduction of this material can effectively improve the maneuverability properties of bone cement. In their study, Li et al. used 15.0% by weight-impregnated MC-PMMA; this material significantly reduced the modulus of elasticity of PMMA bone cement from 1.91 to 1.21 GPa (Li et al., 2015). The results also revealed that the addition of MC significantly reduced the compressive elastic modulus of PMMA, thereby reducing the pressure on adjacent vertebrae. However, addition of MC had no significant effect on the injectibility and processing time of the cement. Similarly, Zhu et al. found that the addition of MC improved the handling properties of this composite bone cement in the clinical setting (Zhu et al., 2020) (Figure 5). Clinical evidence suggests that patients treated with MC-PMMA show significant improvement in postoperative low back pain, dyskinesia, and vertebral height (Bai et al., 2017). Notably, patients in a study who were treated with MC-PMMA demonstrated greater improvements in bone density at 6-month and 1-year follow-up than those treated conventionally; the incidence of adjacent vertebral fractures decreased to 2% after modified cementing. This represented a significant improvement over the rate of 13% observed after conventional cementing (Wang et al., 2018a).

#### 3.4.2 Synthetic polymers

**FIGURE 6:**
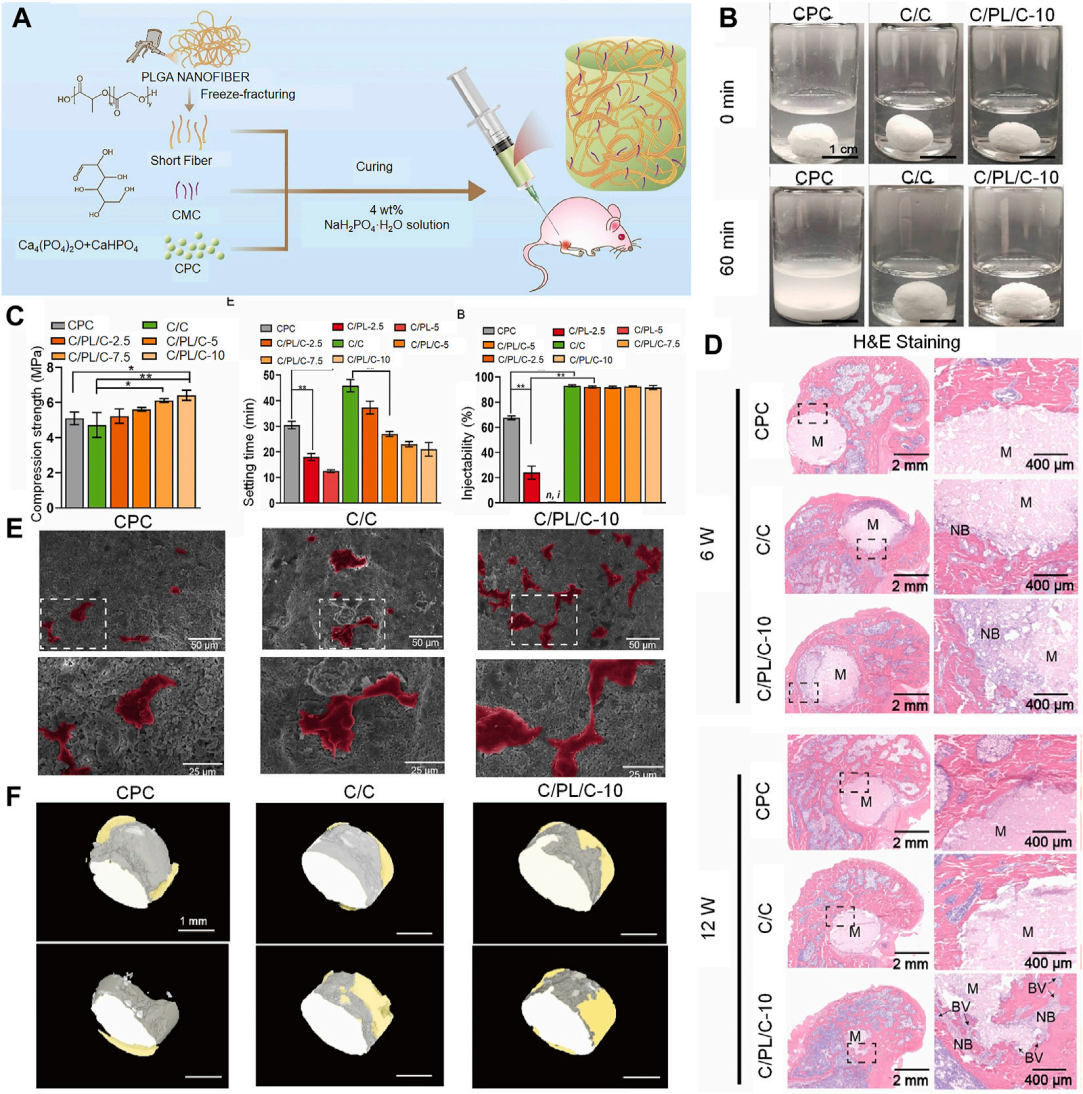
Injectable PLGA nanofiber-reinforced bone cement with controlled biodegradability for bone regeneration after minimally-invasive surgery. **(A)** Schematic representation of fabrication of C/PL/C injectable bone cement for bone regeneration. **(B)** Anti-washout performance of CPC, C/C, and C/PL/C-10, respectively. **(C)** Injectability, compressive strength, and setting time of cements. Data are presented as the mean ± standard deviation; n = 3; *significant difference compared with control group, *p < 0.05 and **p < 0.01. **(D)** Hematoxylin and eosin staining of non-decalcified femoral condyle sections 6 and 12 weeks after implantation. NB: newly formed bone; BV: blood vessels; M: materials. **(E)** Scanning electron microscope images of human umbilical vein endothelial cells on the cement surface after 24 h of incubation (human umbilical vein endothelial cells are in color for ease of observation). **(F)** 3-dimensional reconstruction of the implants in different groups; gray: cement; yellow: newly formed bone. ^©^ 2022 The Authors. Publishing services by Elsevier B.V. on behalf of KeAi Communications Co. Ltd.

Synthetic polymers offer more possibilities for chemical modifications and molecular alterations than their natural counterparts. This may help tailor system performance to specific application requirements (Wong et al., 2023). These polymers have customized matrix structures and chemical properties. In this context, poly (lactide-co-glycolide) (PLGA) is a linear copolymer of lactic and glycolic acid monomers (Jin et al., 2021). The time needed to degrade PLGA can be adjusted (to align with that of bone regeneration) by adjusting the ratio of lactic and glycolic acid. The degradation of PLGA gradually enhances stress stimulation of new bone; it may therefore promote bone regeneration and structural remodeling. PLGA is currently incorporated into bone cement to improve its properties. CPC scaffolds are doped with novel PLGA microspheres; these microspheres provide pores that allow CPC to grow into the new bone tissue. In a study using a rat femoral defect model, the PLGA microspheres were found to be nearly filled with mature new bone upon degradation at 24 weeks (Liang et al., 2020). Composites of dense PLGA particles have also been found to be suitable for use as pore generators in CPC; in a study, they accelerated degradation and were more effective in promoting murine BMSC proliferation (Qian et al., 2020; Lu et al., 2021). In their study, Yu et al. found that the addition of fibrous PLGA to bone defects effectively improved the brittleness of CPC. A two-fold increase in toughness was observed in addition to a moderate improvement in compressive strength (Yu et al., 2018; Cai et al., 2023) (Figure 6).

PLGA is effective in improving the handling properties of bone cement; it has also been widely used as a drug delivery system for molecules such as proteins, peptides, and genes due to its excellent carrier properties. PLGA was first used in a study on the treatment of infectious osteomyelitis; it was used in the form of microspheres after immobilizing ciprofloxacin and triclosan-containing PLGA microspheres on PMMA (Wang et al., 2020b). In their study, Qiao et al. loaded rifampicin/moxifloxacin onto PLGA microspheres for local drug delivery; microspheres prepared using PLGA and embedded with moxifloxacin and rifampicin/moxifloxacin using the water-in-oil-in-water double emulsion solvent evaporation technique were used for local delivery (Qiao et al., 2019). The results from these studies provide valuable insights into the treatment of osteoporotic and neoplastic VCFs. PLGA can also be used as a drug carrier for the treatment of oncologic bone disease. In their study, Jayaram et al. added zoledronic acid to PLGA, which released it at a concentration of 8% over 97 weeks. In contrast, PMMA released 13%–17% of zoledronic acid; PLGA therefore offered better release kinetics (Jayaram et al., 2021). PLGA loaded with doxorubicin was combined with bone cement in a study; it prolonged the release of doxorubicin and has a positive impact on the treatment of sarcoma (Dewhurst et al., 2020). In the treatment of osteoporotic VCFs, nano-sized PLGA particles were able to encapsulate and release the functional recombinant protein (ICOS-F) into cement formulations to provide an anti-osteoclastic effect and stimulate an appropriate bone remodeling response, which was conducive to effective healing (Banche-Niclot et al., 2023). On injecting CPC/PLGA composites loaded with alendronate in an osteoporotic rat model, the composites exhibited a suitable setting time, appropriate compressive strength, and controlled release of alendronate; bone formation was also demonstrated under osteoporotic conditions (van Houdt et al., 2018).

PVA fibers have been added to other gel matrices (as high-tenacity materials) due to their excellent high modulus of elasticity and tensile strength (Shi, 2021). The area of fiber reinforcement of cement matrices (used in civil engineering) has been researched extensively. However, the findings have been less frequently applied to medical bone cementing. It is believed that PVA fibers are usually covered by a hydrophobic oil-based coating, which reduces their hydrophilicity and optimizes energy dissipation via a friction-sliding mechanism; this hydrophobic PVA improves toughness and ductility (Kucko et al., 2019). In view of its high toughness, it may be a good candidate for incorporation into brittle bone cements such as CPC. In the dental field, reinforcement of the cement matrix with PVA fibers has led to the successful development of tough fiber-reinforced CPCs. The incorporation of PVA fibers reinforces CPCs to improve cement toughness and structural stability upon degradation; however, it does not affect biocompatibility and the osseointegration process (Schickert et al., 2020a). Reinforcement with PVA fibers increases the flexural strength and toughness of CPCs by more than 3 and 435-fold, respectively; this makes reinforcement an extremely effective strategy for strengthening and toughening (Kucko et al., 2019). In their study, Luo et al. found that cement containing 5 percent by weight of fibers offer a good compromise, with compressive strengths of 46.5 ± 4.6 MPa (compared to 62.3 ± 12.8 MPa without fibers), which are considerably greater than that of human trabecular bone (0.1–14 MPa) (Luo et al., 2019).

## 4 Vertebral implants

**FIGURE 7:**
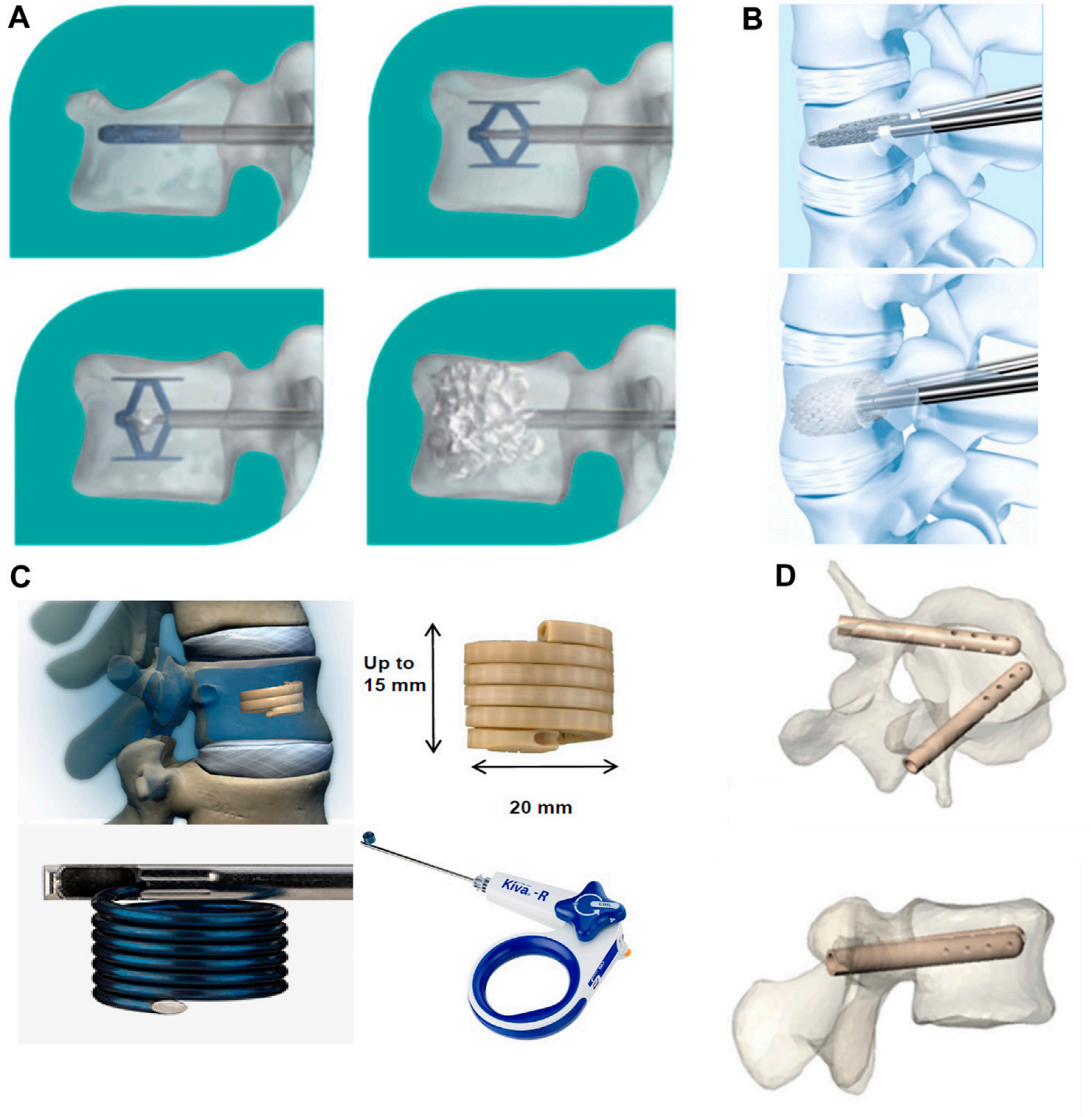
Innovative spine implants for improved augmentation and stability in neoplastic vertebral compression fractures. **(A)** SpineJack^®^ implantation procedure. **(B)** Vertebral Body Stent^®^ (VBS^®^) deployment procedure. **(C)** KIVA^®^ implant design and delivery ancillaries. **(D)** Views of V-STRUT^©^ implants in a vertebra, perspective and top view. ^©^ 2019 The Authors. Published by MDPI.

Unlike minimally invasive injectable cement materials, vertebral implants are primarily composed of implantable metals (Cornelis et al., 2019) (Figure 7). These include the Vertebral Body Stenting (VBS), SpineJack, Kiva, and Osseofix systems, which are based on a similar principle of percutaneous implantation of an expandable vertebral body stent (to restore vertebral height) and the correction of kyphosis; this procedure is referred to as third-generation vertebral body augmentation (Dong et al., 2022).

The goal of restoring mechanical stability to the diseased vertebral body is achieved by use of a vertebral body implant. An *in vitro* biomechanical study on the Osseofix system used human cadaveric vertebrae; it showed that the yield and ultimate loads of the vertebrae repaired by the system were similar to those of intact vertebrae. In addition, the Osseofix system was effective in restoring the original biomechanical strength of fractured vertebrae, unlike kyphoplasty (Ghofrani et al., 2010). An *in vitro* study that compared the mechanical properties of the SpineJack system and balloon kyphoplasty in human cadaveric bone found that both procedures restored height; strength and stiffness were partially restored without any significant differences. Although the mechanical properties of most vertebral implants have been well documented, further investigation is needed to assess the clinical effectiveness and scope of application of these metallic implants in posterior convex VCFs (Sietsma et al., 2009). Certain recent clinical studies have found this new type of implant to be effective in the treatment of traumatic VCFs or pathological fractures caused by osteoporosis or metastatic tumors of the spine. A study evaluated the extent of height recovery offered by the VBS in cases of acute traumatic VCFs among young non-osteoporotic patients. The values for mean postoperative vertebral height gain, vertebral kyphosis angle correction, and Beck index improvement were 3.8 mm, 4.3°, and 0.07, respectively. The results from the study confirmed that VBS can significantly restore vertebral height in young patients with traumatic VCFs (Garnon et al., 2019). A study had retrospectively evaluated the safety and efficacy of the VBS in patients with post-traumatic A3.2 and A2 type fractures (single traumatic thoracolumbar fractures) who were treated between 2010 and 2019. The results confirmed an improvement in posterior convexity and restoration of vertebral height in all patients (Salle et al., 2022). In a prospective study on type A1.3 and A3.1 fractures, the VBS offered satisfactory improvements in pain, function, posterior convexity correction, and even endplate repositioning in osteoporotic and traumatic fractures (Klezl et al., 2011). The system was also found to be effective in correcting kyphotic deformities and restoring loss of vertebral height in patients with chronic osteoporosis who had VCFs; these findings confirm the feasibility of its clinical application (Premat et al., 2018). A recent study evaluated the utility of the OsseoFix system for the treatment of VCFs caused by multiple myeloma; it found that the implant provided significant improvements in terms of both pain and prognostic scores, thereby significantly reducing complications. The total number of implants used in this study was the highest to be reported in the literature to date; the use of expandable titanium mesh cages allowed safe and effective treatment (Gandham et al., 2021).

Although, new implant materials have been able to address certain limitations of PVP and PKP, various clinical adverse events continue to occur. For instance, stent tumbling prevents the contralateral stent from providing adequate support (Kanematsu et al., 2023). In this context, a randomized controlled trial evaluated the impact of two different augmentation procedures, namely, the KIVA system and PKP, on the readmission rate due to serious adverse events. The patients with a previous history of VCF or significant osteoporosis who were treated using the KIVA system demonstrated a greater risk of readmission due to serious adverse events (at 1-year after treatment) than those who underwent PKP (Beall et al., 2017). Therefore, future studies need to evaluate the issue of appropriate selection of metallic implants for different vertebral body fractures.

## 5 Conclusion and prospects

The appropriate selection of implantable materials is crucial to the clinical outcomes of minimally invasive treatment for VCFs. However, the materials in current use have various limitations, which hinder clinical application. As the first and second generation of minimally invasive implantable materials for vertebral body augmentation, traditional bone cement (represented by PMMA) is widely used in the clinic. However, it lacks bioactivity and leads to a series of clinical complications. Therefore, newer bone cements including MPC and CSC have been developed; these have been favored by researchers owing to their superior osteoclastogenic and angiogenic effects. Biomaterials with various beneficial properties have been mixed with bone cement to improve its handling properties and bioactivity in a targeted manner. A review of the types and clinical efficacy of new vertebral implants used in third-generation vertebroplasty show that a wide variety of options are available for the treatment of different types of VCFs.

Although the implant materials developed for minimally invasive treatment have various outstanding properties, their clinical effectiveness and safety remain unclear. In addition, clinical validation of processing properties such as injectability and setting time are lacking for new composite bone cement materials. Large-scale controlled clinical studies evaluating the efficacy of new vertebral implants are also lacking; the scope of their application warrants further investigation. Clinicians also need to address the issue of selection of appropriate materials for minimally invasive surgery. In conclusion, the ongoing improvements in technology and biomaterials are expected to make minimally invasive surgery for VCFs safer and more effective.
